# Eyebrow hairs from actinic keratosis patients harbor the highest number of cutaneous human papillomaviruses

**DOI:** 10.1186/1471-2334-13-186

**Published:** 2013-04-24

**Authors:** Ines Schneider, Mandy D Lehmann, Vlada Kogosov, Eggert Stockfleth, Ingo Nindl

**Affiliations:** 1 Charité, Department of Dermatology, Venereology and Allergy, Skin Cancer Center Charité, University Hospital of Berlin, Berlin, Germany; 2 DKFZ – Charité, Viral Skin Carcinogenesis Group, Division Viral Transformation Mechanisms, German Cancer Research Center (DKFZ), Heidelberg, Germany; 3 Charité - Universitätsmedizin Berlin, Klinik für Dermatologie, Venerologie und Allergologie, Charitéplatz 1, Berlin, 10117, Germany

**Keywords:** Actinic keratosis (AK), Cutaneous human papillomavirus (HPV), Eyebrow hairs, Natural history

## Abstract

**Background:**

Cutaneous human papillomavirus (HPV) infections seem to be associated with the onset of actinic keratosis (AK). This study compares the presence of cutaneous HPV types in eyebrow hairs to those in tissues of normal skin and skin lesions of 75 immunocompetent AK patients.

**Methods:**

Biopsies from AK lesions, normal skin and plucked eyebrow hairs were collected from each patient. DNA from these specimens was tested for the presence of 28 cutaneous HPV (betaPV and gammaPV) by a PCR based method.

**Results:**

The highest number of HPV prevalence was detected in 84% of the eyebrow hairs (63/75, median 6 types) compared to 47% of AK lesions (35/75, median 3 types) (p< 0.001) and 37% of normal skin (28/75, median 4 types) (p< 0.001), respectively. A total of 228 HPV infections were found in eyebrow hairs compared to only 92 HPV infections in AK and 69 in normal skin. In all three specimens HPV20, HPV23 and/or HPV37 were the most prevalent types. The highest number of multiple types of HPV positive specimens was found in 76% of the eyebrow hairs compared to 60% in AK and 57% in normal skin. The concordance of at least one HPV type in virus positive specimens was 81% (three specimens) and 88-93% of all three combinations with two specimens.

**Conclusions:**

Thus, eyebrow hairs revealed the highest number of cutaneous HPV infections, are easy to collect and are an appropriate screening tool in order to identify a possible association of HPV and AK.

## Background

Human papillomaviruses (HPV) are non-enveloped double-stranded DNA viruses that infect epithelial cells. Presently, 151 HPV types are completely characterized (http://pave.niaid.nih.gov) and classified into mucosal/genital (alphaPV) and cutaneous (betaPV, gammaPV, muPV, and nuPV) HPV types, based on sequence analyses and clinical manifestation [1,2]. Their causal association with (ano)genital carcinoma is fully established [3,4]. The increased risk of cutaneous squamous cell carcinoma (SCC) in the presence of HPV infections of immunocompetent as well as immunosuppressed patients enlarged the interest in the role of cutaneotropic HPV in skin cancer [5-7]. Several studies showed oncogenic potential of cutaneous HPV types with both *in vitro* (different cell lines) and *in vivo* models (animals) [8-15]. The molecular mechanisms of beta1PV (e.g., HPV5, HPV8, and HPV20) are very likely different from beta2PV (e.g. HPV23 and HPV38) [8,10,15]. At present, HPV38 is the only cutaneous type, which is able to immortalize human primary keratinocytes [10] indicating that the oncogenic potential of beta2PV seems to be higher compared to the beta1PV types. However, to proof the causal role of HPV and skin cancer, 4 major criteria have to be fulfilled; i) and ii) presence and activity of HPV, iii) transforming properties and iv) increased risk in epidemiological studies.

The first evidence for the association of HPV in the development of actinic keratosis (AK) and cutaneous SCC was shown in patients with the disease Epidermodysplasia verruciformis (EV) [16,17]. EV patients suffer a rare genetic defect that results in the formation of macular lesions, which frequently progress to SCC [18]. In the SCC lesions of EV patients, cutaneous HPV types (predominantly beta1PV types especially HPV5 and HPV8) have been detected and are very likely etiologically linked with skin cancer in this specific genetic background [19], which is supported by HPV expression in skin tumors of EV patients [20].

In SCC of non-EV patients both beta1PV and beta2PV were detected [7,21]. In large epidemiological studies HPV23 was the most prevalent type in the normal population as well as in immunosuppressed and immunocompetent skin cancer patients [5,7,22]. However, in contrast to genital HPV, no specific HPV type has been associated with an increased risk of skin cancer. The estimated relative risk (odds ratio) of SCC increased with the number of cutaneous HPV types and was highest in biologically active infections (presence of both antibodies and DNA for at least one HPV type) [5,7]. Moreover, not all dysplastic cells are infected with cutaneous HPV resulting in a low viral load. Only one of approximately 100–1,000 skin tumor cells are HPV infected with a higher viral load in AK compared to SCC [23]. In summary, it is very likely that HPV types are a co-factor in association with UV-radiation in the early pathogenesis of cutaneous SCC [24-26].

So far, only little is known about the natural history of cutaneous HPV types and in previous epidemiological studies mostly eyebrow hairs, skin swabs and rarely normal skin samples have been used to determine the cutaneous HPV profile of patients or volunteers [27-29]. Cutaneous HPV types are present in hair follicles [27] with a high concordance of different non-genital body sites [30]. Hair follicles are very likely the natural reservoir of cutaneous HPV types and are easy to collect. Therefore, they may be a useful noninvasive marker of monitoring cutaneous HPV infections. However, whether eyebrow hairs or normal skin should be used to examine cutaneous HPV infections of AK patients is still unknown. In our study, we analyzed cutaneotropic HPV types in eyebrow hairs, normal skin and AK lesions to identify the optimal specimen in monitoring cutaneous HPV infections.

## Methods

### Study population

This study was designed according to a previously reported study analyzing the tumor-specific mutations of p53, p16^INK4a^ and Ha-Ras in immunocompetent AK patients [31]. Briefly, cryo-biopsies of 75 AK patients (aged 57–88 years old, median 71) were taken from the scalp (sun-exposed, low-graded AK lesions) and the inner site of the upper arm (sun-shielded, normal skin) at the Charité in Berlin. Normal skin and AK lesions were placed in liquid nitrogen within 2 min after resection and stored at −70°C. We have performed frozen sections of all divided AK lesions to confirm the presence of dysplastic cells before we have isolated the DNA. The other half of each AK tumor biopsy was fixed in formalin and embedded in paraffin for histological evaluation. Moreover, 8–10 eyebrow hairs were plucked from each AK patient and stored at −70°C until processing. The study was approved by the local ethics committee at the Charité, University Hospital Berlin, Berlin, Germany (number Si. 248) and was conducted according to the Declaration of Helsinki. All patients gave written consent.

### Cutaneous HPV detection and genotyping

DNA isolation of both cryo-biopsies and eyebrow hairs was performed using QIA-amp DNA Mini Kit (Qiagen; Hilden Germany) according to the manufacturer’s protocol. The samples were eluted in 100 μl of AE buffer and stored at −20°C until processing. HPV detection and typing was performed as previously described [32,33]. With this consensus primer-mediated PCR method, we were able to detect 25 beta and 3 gamma cutaneous HPV types (BGC-PCR) amplifying a 72-bp L1 fragment. Briefly, each PCR included three negative controls (water), three positive controls containing 10, 100, or 1000 copies of HPV8 plasmid DNA in a background of 100ng human placental DNA per reaction and 10ng/μl template DNA. Genotyping was performed with 28 HPV specific 5-amino-linked oligonucleotides by reverse-line-blotting (RLB). The quality of each clinical specimen was examined by β-globin PCR, as described previously [34], and only PCR positive specimens were included in the present study. All clinical specimens were analyzed twice on different days, and HPV types were only scored if both experiments revealed consistent results. The reproducibility of both experiments was very high and similar as previously described [32].

### Statistical analyses

**Figure 1:**
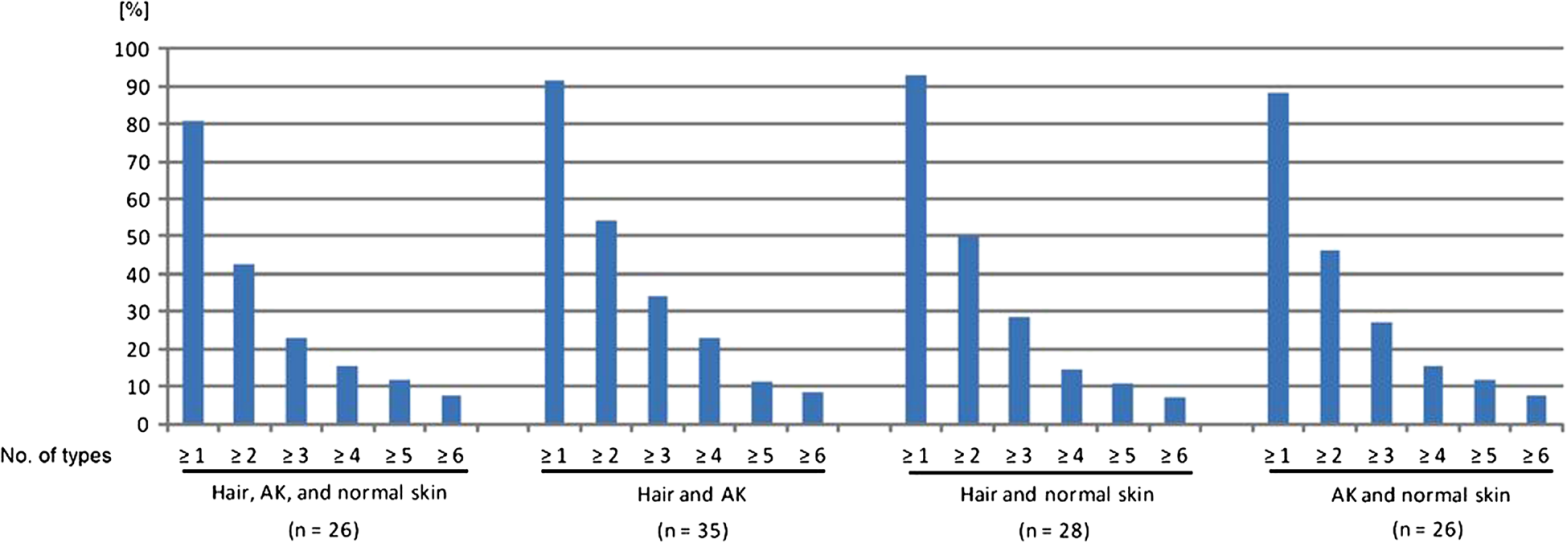
**Concordance of HPV infections.** Concordant infections of HPV positive specimens with at least 1–6 cutaneous HPV types in eyebrow hairs, actinic keratosis (AK), and normal skin are presented. The overall agreements of four combinations are shown with different definitions of concordance. Specimens were classified as concordance if they contained the same HPV of at least one HPV type, two types et cetera. The overall HPV agreement of at least one HPV type was 81-93%, and of at least two HPV types was 42-54% of all 4 combinations, respectively. The kappa values calculated with all 75 specimens of at least one (≥1) and two (≥2) type(s) in common of hairs versus AK was 0.314 (95% CI: 0.167-0.460) and 0.321 (95% CI: 0.178-0.463) (fair agreement), hairs versus normal skin was 0.217 (95% CI: 0.099-0.335) and 0.259 (95% CI: 0.129-0.390) (fair agreement), and AK versus normal skin was 0.692 (95% CI: 0.527-0.856) and 0.621 (95% CI: 0.410-0.833) (good agreement). No., numbers.

The prevalence of cutaneous HPV in each specimen (eyebrow hairs, biopsies of AK and normal skin) was calculated. A specimen was rendered positive if at least one HPV type was detected and statistical analysis was performed using chi-square testing or fisher-exact testing, when frequencies were smaller than five. A p-value of < 0.05 was considered significant either for alpha, or for alpha multiple with p-adjustment. HPV concordance between any two sample types of the three different specimens (eyebrow hairs, AK, and normal skin) was calculated with the U-test by Wilcoxon, Mann, and Whitney (Figure 1). Kappa-values express the proportion of possible agreement beyond chance. A kappa estimate of less than 0.4 represents poor agreement, a kappa estimate between 0.4 and 0.75 correlates fair to good agreement, and a kappa estimate of more than 0.75 indicates excellent agreement.

## Results

### HPV infections in three different specimens

**Figure 2:**
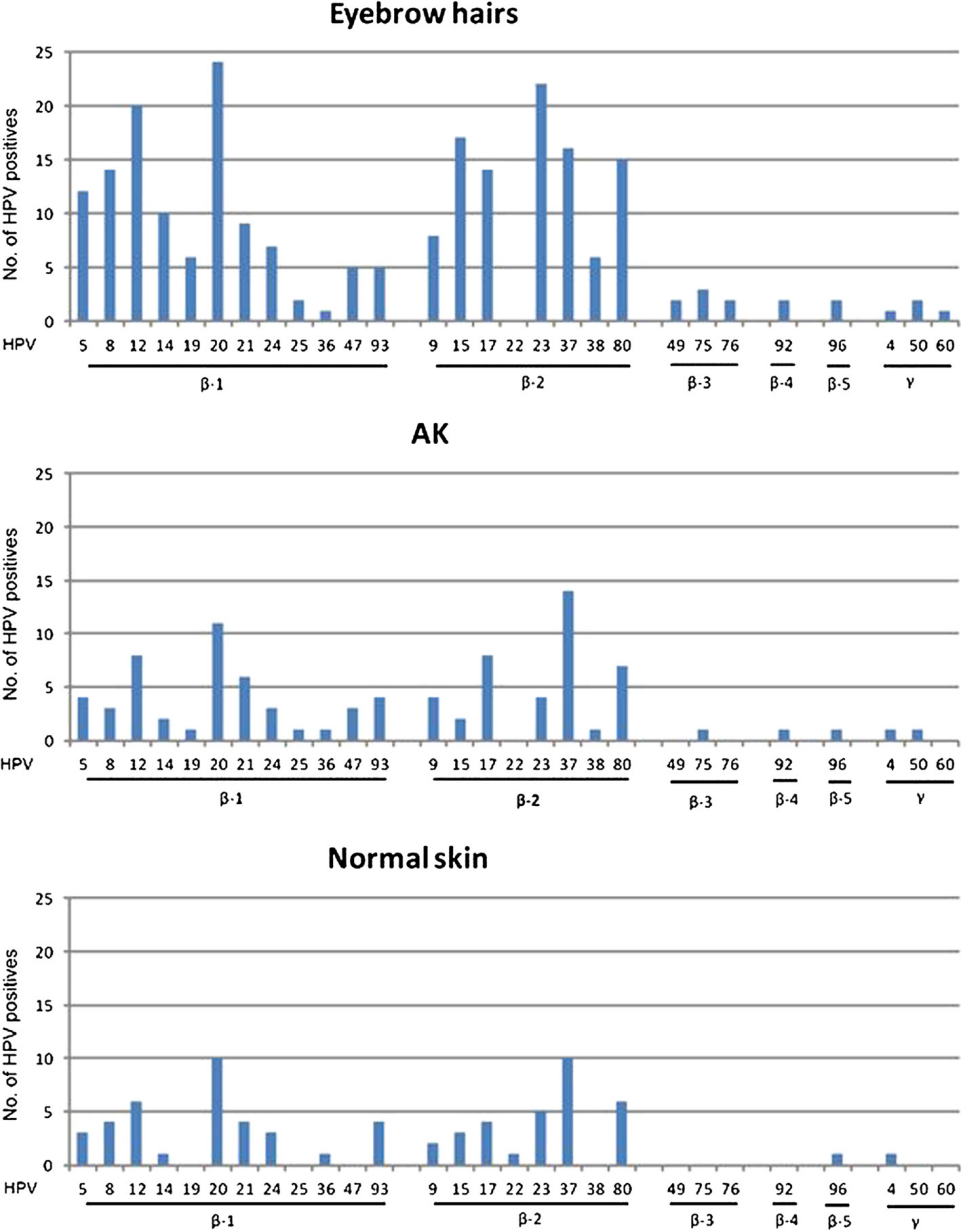
**Prevalence of cutaneous HPV types.** HPV infections of all 28 examined cutaneous HPV types (beta-1, -2, -3, -4, -5, and gamma PV) in three different specimens of 75 AK patients. The total of HPV infections was found highest in eyebrow hairs (228) compared to significantly lower viral infections in AK (92) and in normal skin (69). No., numbers.

**Figure 3:**
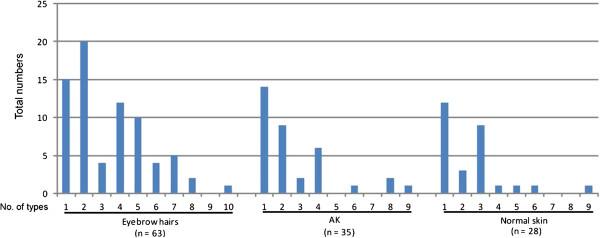
**Muliplicity of HPV infections.** The percentages of single (1 type) and multiple infections (2–10 types) of HPV positive eyebrow hairs, actinic keratosis (AK) and normal skin are shown. The highest number of multiple infections was observed in 76% of the eyebrow hairs (48/63; median 6 types), compared to lower numbers of 60% in AK (21/35; median 3 types), and of 57% in normal skin (16/28; median 4 types), respectively.

A significant higher number of cutaneous HPV infections (betaPV and gammaPV) were detected in eyebrow hairs (63/75; 84%) compared to AK lesions (35/75; 47%) and normal skin (28/75; 37%) (p<0.001), respectively. All 28 HPV types examined were detected in the present cohort (Additional file 1: Table S1). The following HPV types were not detected in eyebrow hairs (HPV22), in AK (HPV types 22, 49, 60, and 76), and in normal skin (HPV types 19, 25, 38, 47, 49, 50, 60, 75, 76, and 92) (Figure 2). Only HPV49, HPV60 and HPV76 were exclusively found in eyebrow hairs, however with a low total number of HPV types (1–3). Thus, no specific HPV type seems to be associated with AK or the control group (hairs and/or normal skin). The most prevalent HPV types were HPV12, HPV15, HPV20, HPV23, and HPV37 in eyebrow hairs (at least 15/75), and HPV20 and HPV37 in both AK and normal skin (at least 10/75) (Figure 2). A high prevalence of HPV20 and HPV37 was found in all three specimens, and thus these HPV types were not AK specific. The presence of a single HPV type was nearly two-fold higher in AK and normal skin (40% and 43%) versus eyebrow hairs (24%) resulting in a high number of multiplicity in eyebrow hairs (Figure 3). With other words, multiple infections were found in 76% of the eyebrow hairs, compared to a lower number of 60% in AK and 57% in normal skin. From the HPV positive specimens multiple infections with at least 5 HPV types were present in 35% of eyebrow hairs (22/63) compared to only 11% in both AK (4/35) and normal skin (3/28) (Figure 3). In eyebrow hairs, up to 10 different HPV types were present in a single specimen (median 6 types). In AK and normal skin up to 9 types were found with a lower median number of HPV types (median 3 and 4 types, respectively). Overall, HPV prevalence, multiple infections and the number of different HPV types present in a single specimen were highest in eyebrow hairs compared to lower numbers in biopsies of AK lesions and normal skin.

### Concordance of HPV infections in eyebrow hairs, normal skin and AK

We defined concordant infections with different stringencies of at least one and up to six HPV types present in two or three different specimens of one patient. In this analysis only HPV positive specimens were included. Overall cutaneous HPV agreement of at least one HPV type was between 81-93% in all four combinations of three samples (Figure 1). The highest number of concordant infections with at least one HPV type was found in eyebrow hairs compared to AK (91%, 32/35) or to normal skin (93%, 26/28). A marginal lower number of 88% was observed in AK lesions compared to normal skin. In eyebrow hairs, AK and normal skin 81% of the specimens were infected with the same HPV of at least one type. Using a more stringent definition of concordance with the same HPV infection of at least two types, the concordant infections decreased to 54% (hairs and AK), 50% (hairs and normal skin), 46% (AK and normal skin), and 42% (hairs, AK, and normal skin), respectively. In a direct comparison of all 75 specimens, only fair agreements were found between hairs versus AK or normal skin (kappa values of 0.314 or 0.217). The highest kappa value of 0.692 (95% CI: 0.527-0.856) was estimated in AK versus normal skin (Figure 1), which express a good agreement. The kappa values show the high HPV concordance in biopsies (AK lesions and normal skin), which differ in hairs due to the higher number of HPV infections.

## Discussion

Eyebrow hairs are infected with cutaneous HPV types, they are easy to collect and often used in epidemiological studies as noninvasive markers of HPV infections. Alternatively, instead of eyebrow hairs perilesional tissue or normal skin can be used as a control and reference of skin cancer. Both specimens are difficult to collect, only rarely used and normally not available especially in large epidemiological studies. Currently it is unknown whether normal skin tissue or eyebrow hairs best represent the cutaneous HPV status of AK patients. In our present comprehensive study, we evaluated the prevalence and the concordant infections of cutaneous HPV types in eyebrow hairs and normal skin compared to skin lesions from 75 AK patients. We identified that eyebrow hairs contained the highest number of different HPV types.

The viral load of cutaneous HPV types in eyebrow hairs, normal skin, AK and cutaneous SCC is normally very low [23,35,36]. Only one of 100–1,000 cells are infected with cutaneous HPV types but the number of multiple infections is very high [26]. Thus, methods with a high sensitivity and a large spectrum of types are required to detect all cutaneous HPV types that are present in humans.

Eyebrow hairs were collected from 126 AK patients, and cutaneous HPV types were detected in 54% of hairs by a type specific PCR method able to detect six HPV types [37]. We found more cutaneous HPV types and a higher number of multiple infections in hairs of AK patients. The difference is most likely due to the limited number of HPV types detected compared to 28 types examined in our study. Eyebrow hairs of 171 participants were examined to calculate the risk of betaPV infections of AK [38]. Any betaPV type was detected in 73% of healthy individuals, and in 70-72% of AK patients, which was similar with 84% in hairs of AK patients of our study.

De Koning and colleagues examined eyebrow hairs from 845 healthy individuals (SCC-free) from six different countries and cutaneous HPV infections were detected in 84-91% [22]. Another study analyzed eyebrow hairs of 845 healthy humans without SCC from three countries and cutaneous HPV types were found in 89% [7]. Thus, the percentage of cutaneous HPV types in eyebrow hairs of healthy individuals was high and comparable with AK patients.

Only few studies examined cutaneous HPV types in normal skin biopsies, because routinely only perilesional skin is removed during surgery of skin cancer. A low number of cutaneous HPV was found in 16% of normal skin (57 skin biopsies) [21]. In a further study 72 normal skin samples were collected and typed for cutaneous HPV by a degenerated nested PCR technique and HPV prevalence in these lesions was 18% [39]. In our study, a higher prevalence of cutaneous HPV in normal skin was found.

In a recent comparative study cutaneous HPV infections of 21 SCC patients were examined with four different specimens; eyebrow hairs, perilesional skin, normal skin and SCC [40]. A high overall HPV prevalence was detected in every specimen (81% to 95%), and a significantly higher median number of HPV types in SCC (6 types), perilesional skin and eyebrow hairs (5 types) compared to normal skin (2 types). In our study, we observed a lower HPV prevalence in normal skin (37% versus 81%) but a similar high prevalence in eyebrow hairs. We have collected normal skin biopsies from the inner site of the upper arm from 75 AK patients compared to normal skin from the mirror image site of 21 SCC patients. Both PCR based methods detect the same beta HPV types and have a comparable sensitivity, thus the different methods used in both studies are very unlikely responsible for the difference. Another explanation is the different cohort (AK in our study versus SCC patients in the other study).

Cutaneous HPV infections were found in 41% of AK (27 skin biopsies) by PCR based methods able to detect approximately 100 HPV types [21]. We detected a similar number of cutaneous HPV types in skin lesions from 75 AK. In another study a higher prevalence of cutaneous HPV types (85%) was found in 54 AK lesions by a nested PCR [41]. The different prevalence rates in skin lesions (85% versus approximately 50%) may be due to a higher sensitivity of nested PCR versus single PCR, the different cohorts or the low number of specimens examined. Further studies have to be performed to estimate the accurate HPV prevalence in AK lesions.

## Conclusions

In our study, not a specific type or particular combinations of HPV types were detected in AK lesions compared to normal skin and eyebrow hairs. The concordance of all three different specimens of HPV positive samples was high. Most importantly, we identified a significant higher number of cutaneous HPV types in eyebrow hairs compared to normal skin and skin lesions of AK patients. Thus, eyebrow hairs seem to be an appropriate marker to examine the role of cutaneous HPV and AK.

## Competing interests

The authors declare that they have no competing interest.

## Authors’ contributions

IS analysed the data, created the figures and revised the manuscript. MGL performed all experiments. VK interpreted the results and revised the manuscript. ES collected all clinical specimens, interpreted the results and revised the manuscript. IN designed the study, monitored the project activities and coordinated the activities of the laboratory, interpreted the results, drafted and edited the manuscript. All authors read and approved the final manuscript.

## Pre-publication history

The pre-publication history for this paper can be accessed here:

http://www.biomedcentral.com/1471-2334/13/186/prepub

## Supplementary Material

Additional file 1: Table S1 Cutaneous HPV types detected in 3 different specimens of 75 immunocompetent AK patients. Click here for file
