# Supplementary material for: Eyebrow hairs from actinic keratosis patients harbor the highest number of cutaneous human papillomaviruses
Source: BMC Infect Dis. 2013 Apr 24;13:186. doi: 10.1186/1471-2334-13-186 (PMC3642014; doi:10.1186/1471-2334-13-186)
Supplement: Additional file 1: Table S1 — Cutaneous HPV types detected in 3 different specimens of 75 immunocompetent AK patients. [file 1471-2334-13-186-S1.pdf]

Table S1: Cutaneous HPV types detected in 3 different specimens of 75 immunocompetent AK patients

Patient Number	Sex	Age (years)	HPV types in eyebrow hairs	HPV types in AK lesions	HPV types in normal skin
1	m	62	neg	neg	neg
2	m	69	8	neg	neg
3	m	81	8,12	neg	neg
4	m	83	5,8,12,17,20,21, 24,37,76,93	5,17,19,20,21,24 36,37	5,8,12,17,20 21,24,36,37
5	f	73	12,24,37,80	37	23
6	f	73	5	neg	neg
7	f	72	neg	neg	neg
8	m	66	8, 20	neg	20
9	m	67	9,17,75	neg	neg
10	m	68	5, 15, 37, 80	37,80	neg
11	m	57	neg	neg	neg
12	m	76	5	neg	neg
13	m	71	4,5,8,17,20,23	4,20	4,15,20
14	m	67	8,9,12,17,20,23,37	8,9,12,17,20,23, 37,38,50	8,9,12,17,37
15	m	67	neg	neg	neg
16	m	74	20	neg	neg
17	m	85	5, 23	neg	neg
18	f	73	8,12,15,23,80	neg	neg
19	m	63	5,15,23,37,80	15	15,23,37
20	f	63	neg	neg	neg
21	m	66	12,14,17,20,23,37	17,37	37
22	m	76	17	neg	neg
23	m	65	80	80	80
24	m	68	23,37,80,93	37,93	37,93
25	m	65	20	neg	neg
26	m	67	neg	neg	neg
27	m	66	neg	neg	neg
28	m	75	9,12,14,15,19,20,92,96	9,96	21,23,96
29	m	64	8,12,23,37,47	neg	neg
30	m	63	15	neg	neg
31	m	64	14,80	14,80	neg
32	m	61	neg	neg	neg
33	m	73	9,23,36	neg	neg
34	m	60	8,17,23,50,80	8,17,23,80	5,8,80
35	m	60	8,20	neg	neg
36	m	62	neg	neg	neg
37	m	77	5,14,15,17,21,23,80	17	17
38	m	67	17	neg	neg
39	m	62	19,20,38,60	17	20
40	m	83	9,12,20,23	9,12,20,23	9,20
41	m	63	8,15,20,23	20,23	neg

42	m	81	19,23	neg	neg
43	m	67	12	neg	neg
44	m	76	12,23,24,80,93,96	47	23,24,80
45	m	75	9,12,19,37	12,37	neg
46	f	77	neg	neg	neg
47	f	67	15,17,20,38,76	neg	neg
48	f	67	12,23	neg	23
49	m	77	5,8,12,14,17,20,24,37	5,8,12,14,17,20 24,37	5,8,14,17,20,24
50	m	72	15,23,37,80	37	15,22,37
51	m	75	20	neg	neg
52	m	78	14,15,17,23,24	17	neg
53	m	66	12,19,25,37	12,25,37,80	12,37,80
54	f	80	20,21,47,75,92	20,21,47	20,21
55	f	71	neg	neg	neg
56	f	88	5,9,12,14,19,47	5,9,47,93	93
57	m	71	8,12,20,21,23,24,37	12,20,21,24,37,92	12,20,37
58	m	74	37,80	37	neg
59	f	77	5,15,21,24,47	neg	neg
60	m	73	12,14,15,20,23,80,96	neg	neg
61	m	74	9,14,15,38	neg	neg
62	m	76	8,17,20	neg	neg
63	m	70	5,12,14,20,21,50,93	5,12,21,93	12,21,93
64	m	70	15,37,49	37	37
65	m	74	12,15,20,93,80	12,20,80,93	12,20,80,93
66	m	72	23,38,49,80	80	80
67	m	87	15,20,21,38,75	20,21,75	neg
68	m	61	20, 25	neg	neg
69	m	75	47	neg	neg
70	m	66	neg	neg	neg
71	m	72	15,21,38,96	neg	neg
72	m	67	17,23	50	neg
73	f	84	21	21	neg
74	f	70	20	15, 20	20
75	m	67	37	37	37

M, male; f, female; neg, negative.
